# Distinct Transcriptional Programs Underlie Differences in Virulence of Isolates on Host Plants in a Fungal Pathogen, *Colletotrichum gloeosporioides*

**DOI:** 10.3389/fmicb.2021.743776

**Published:** 2021-11-08

**Authors:** Wonsu Cheon, Young Soo Kim, Kotnala Balaraju, Younmi Lee, Hyeok Tae Kwon, Junhyun Jeon, Yongho Jeon

**Affiliations:** ^1^Department of Plant Medicals, Andong National University, Andong, South Korea; ^2^Microbial Research Department, Nakdonggang National Institute of Biological Resources, Sangju, South Korea; ^3^Agricultural Science & Technology Research Institute, Andong National University, Andong, South Korea; ^4^Department of Biotechnology, Yeungnam University, Gyeongsan, South Korea

**Keywords:** cellophane membrane, *Colletotrichum gloeosporioides*, glyoxylate cycle, pathogenicity, progressive symptoms, qRT-PCR, sporulation, static symptoms

## Abstract

Susceptible host plants challenged by fungal pathogens can display different types of lesions, which can be attributed to environmental factors affecting the nature of interactions between the host and pathogen. During our survey of apple anthracnose in Korea, two distinct types of disease symptoms, designated as progressive (PS) and static symptoms (SS), were recognized. PS is a typical, rapidly enlarging symptom of apple anthracnose, while SS is a small, dark speck that does not expand further until the harvesting season. Isolation and genotyping of pathogens from disease lesions suggested that all of them belong to *Colletotrichum gloeosporioides*, a well-known causal agent of apple anthracnose. Two types of isolates were comparable in growth on media, spore germination and appressorium formation, virulence test on fruits at various temperature conditions. Furthermore, they were analyzed at the molecular level by a phylogenetic tree, RNA-seq, and expression of virulence gene. However, the SS isolates were defective in appressorium-mediated penetration into the underlying substratum. RNA-seq analysis of PS and SS isolates showed that distinct transcriptional programs underlie the development of different types of anthracnose symptoms in host plants. One downregulated gene in SS encoded isocitrate lyase is essential for disease development *via* its involvement in the glyoxylate cycle. It partly explains why SS is less virulent than PS on host plants. Overall, our work challenges the traditional view on the development of different lesion types and provides valuable insights into variations that exist in the pathogen population.

## Introduction

The genus *Colletotrichum* contains a large number of species. Many members of this genus are necrotrophic plant pathogens that cause major losses to economically important crops, fruits, vegetables, and ornamentals (Guarnaccia et al., 2021). *Colletotrichum* species are currently listed as the top 10 most important groups of pathogenic fungi because of their scientific and economic impacts (Dean et al., 2012). They typically cause a disease known as anthracnose in a broad range of host plants, including apples, peaches, avocados, strawberries, and peppers (Baroncelli et al., 2017; Zakaria, 2021). Anthracnose disease occurs in both developing and mature plant tissues, resulting in pre- and post-harvest losses (Bailey et al., 1992). Considering that most crops grown worldwide are susceptible to at least one or more species of *Colletotrichum* (Gomes et al., 2021).

*Colletotrichum gloeosporioides* is considered the most prevalent pathogen on apples (Moreira et al., 2019). Currently, *C. gloeosporioides* is recognized as a species complex with several subspecies, such as *C. asianum*, *C. fructicola*, and *C. gloeosporioides* (Weir et al., 2012). The fungus causes and spreads anthracnose disease in warm and humid environments (Saxena et al., 2016). It infects host plants primarily through wounded sites or soft tissue and produces conidia by developing small asexual fruiting bodies called acervuli. Conidia are usually disseminated by rain splash or overhead irrigation, leading to secondary infection of the host plants. Conidium that lands on the host plant germinates and develops appressorium, a specialized infection structure (Deising et al., 2000; Weir et al., 2012). Within the appressorium, enormous turgor pressure accumulates and is used to drive a narrow penetration peg into plant tissue at the base of the appressorium (Eseola et al., 2021). Upon entry into the host plant, the fungus quickly colonizes the plant tissue, eventually leading to tissue necrosis. As colonization continues, sunken and water-soaked spots that rapidly expand on infected plant tissues become visible, which is typically described as anthracnose symptoms (Weir et al., 2012; Boddy, 2016).

However, a large variation exists in the symptoms produced by *C. gloeosporioides* between different hosts (Nguyen et al., 2009). Furthermore, different types of disease symptoms have been recorded, even within a host plant (Kee et al., 2020). Between-host and within-host variations in anthracnose symptom development are usually ascribed to environmental factors based on the famous “disease triangle” concept in plant pathology. The disease triangle concept states that for a disease to occur, a susceptible host plant, a virulent pathogen, and favorable environmental conditions are required, as there is no disease in conditions where any of these three factors is lacking (Scholthof, 2007; Velásquez et al., 2018). The corollary of this is that the interactions between pathogens and plants are dependent on environmental variables, such as temperature and humidity. Under an environmental condition that is not optimal for disease, there would be a deviation from “disease optimum” in terms of both the number and development of disease symptoms.

Apple (*Malus domestica* Borkh.) is one of the most important fruit crops globally. Among the many apple cultivars in Korea, 22 are grown as major cultivars (Ban et al., 2014). Apple production in Korea covers approximately 31,620ha, with a total annual yield of 582,845 tons, of which 60.8% of the total area and 63.9% of the total yield, respectively, are accounted for by apple production in Gyeongbuk Province [Korean Statistical Information Service (KOSIS), 2015]. Currently, the predominant cultivar is “Fuji” (72.4%), which was introduced from Japan in 1967 (Kim et al., 2007). Apple is susceptible to a wide range of diseases affecting fruit quality and yield. Anthracnose, caused by *Colletotrichum* spp., is one of the most important fungal diseases of apple causing significant yield losses to the apple producers in Korea (Oo et al., 2018) and worldwide (Peres et al., 2005). Genus *Colletotrichum* is one of the most important pathogenic fungi causing economically significant diseases on a wide range of subtropical, tropical, and temperate fruit crops (Cannon et al., 2012; Jayawardena et al., 2016). There were a few reports on the existence of two or more different types of diseases caused by different pathogens on the same fruits, for example, a recent study by Khodadadi et al. (2020) reported that two different species of *Colletotrichum*, such as *C. chrysophilum* and *C. noveboracense*, cause bitter rot disease on apple fruits with varying lesion sizes; similarly, a previous report demonstrated that two species of *C. gloeosporioides* and *C. acutatum* caused bitter rot disease on apple fruit (González et al., 2006).

From 2013 to 2015, we surveyed the occurrence of diseases in apple orchards in the northern Gyeongbuk Province of Korea. During our survey, we noticed anthracnose disease symptoms on apples caused by *C. gloeosporioides* presented as two different types on the same apple fruit at various orchards. However, it is not clear why such a difference in symptoms exists. Therefore, in this study, we aim to (i) identify the two types of the pathogens associated with anthracnose symptoms in detail at the molecular and phenotypic levels; (ii) investigate the differences in virulence ability between progressive symptom (PS) and static symptom (SS) isolates of *C. gloeosporioides* that cause anthracnose in apples; (iii) analyze the effect of ethephon treatment and cellophane membrane (CM) on mycelial growth of PS and SS of *C. gloeosporioides*; and (iv) analyze the transcriptome of *C. gloeosporioides* isolates to gain insight into the possible underlying differences in virulence using transcriptome analysis.

## Materials and Methods

### Survey of Major Disease Occurrences on Apple at Various Orchards in the Northern Gyeongbuk Province in Korea

**Figure 1:**
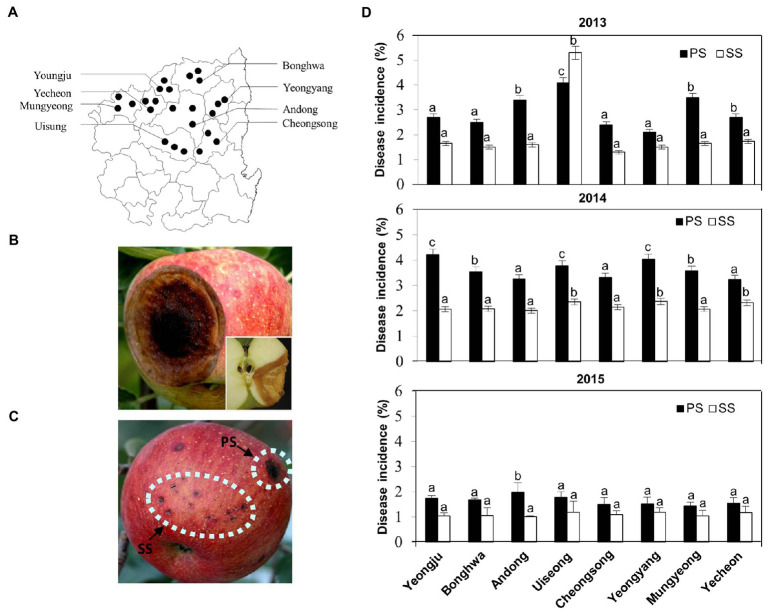
Disease occurrences in apple orchards in Korea over 3years. **(A)** Distribution of disease occurrences in apple orchards at various locations of the northern Gyeongbuk Province of Korea. **(B)** Typical progressive symptoms (PS) of anthracnose appear as round, brown, shrivelled, and sunken spots, with orange conidial masses on the surface of the matured fruits. A longitudinal section of the fruit shown at the corner represents the disease spread as “V” shape inside the fruit. **(C)** An apple fruit showing both types of progressive symptoms (PS) and static symptoms (SS) of anthracnose disease caused by *Colletotrichum gloeosporioides* on the apple fruit in field conditions. **(D)** Occurrence of disease incidence (%) of anthracnose as PS and SS on apple fruits at different locations in the northern Gyeongbuk Province of Korea over 3years, 2013, 2014, and 2015. From each location, 20 samples were collected. Bars with the same letters do not differ statistically from each other, according to the least significant difference (LSD; *p*<0.05).

A survey was conducted over 3years (from 2013 to 2015) on the occurrence of major apple diseases at several apple orchards (Youngju, Bonghwa, Andong, Uiseong, Cheongsong, Yeongyang, Mungyeong, and Yecheon) in the northern Gyeongbuk Province of Korea. For the disease survey, three apple orchards were randomly selected per location (Figure 1A). The locations were as follows: Chunyang-myeon, Bongsung-myeon and Beopjen-myeon from Bonghwa; Sunheung-myeon, Bonghyun-myeon, and Anjeong-myeon from Youngju; Yongmun-myeon, Yecheon-eup, and Gamcheon-myeon from Yecheon; Mungyeong-eup, Maseong-myeon, Sanbuk-myeon from Mungyeong; Angye-myeon, Bian-myeon, and Bongyang-myeon from Uisung; Hyeonseo-myeon, Budong-myeon, and Pacheon-myeon from Chungsong; Bukhu-myeon, Waryong-myeon, and Imha-myeon from Andong. Disease occurrence was recorded in three main apple cultivars: Fuji, Yoko, and Hongro during the months of late June to early October at 20-day intervals. For the disease survey analysis, 25 trees were randomly selected from each orchard and the percentage (%) of diseased fruits was recorded for anthracnose based on the disease index of each plant. The diseases were identified based on diagnostic symptoms following the guidebook by Choi et al. (2012). The common name and scientific name were written based on the list of plant diseases in Korea (The Korean Society of Plant Pathology, 2009).

### Isolation of Pathogenic Fungi From the Apple Orchards and Microscopy

The progressive (PS) and static (SS) pathogenic fungal isolates of pathogen *C. gloeosporioides* used in this study were originally isolated from “Fuji” apples in Korea. The fungi were maintained on potato dextrose agar (PDA; Difco, United States) at 25°C. To prepare spore suspensions, symptomatic tissues were cut from the apple fruits and subjected to surface sterilization using 1% sodium hypochlorite (NaOCl) solution for 1min and 70% ethanol for 30s and then rinsed twice with sterile distilled water (SDW). After sterilization, the tissues were dried on sterile filter paper, placed onto PDA plates, and incubated at 25°C for 7days. Conidial suspensions of pathogenic fungi were prepared by suspending mycelia scraped from a 7-day-old PDA culture plate with SDW. The resulting suspensions were filtered through double-layered cheesecloth, and their concentrations were adjusted to 10^5^ conidia/ml using a hemocytometer (Eckert and Brown, 1986). For microscopic analysis, conidia from the mycelial growth of the fungi were mounted on glass slides and observed under a ProgRes speedXT ^core^3 Imager microscope using differential interference contrast illumination.

### Molecular Identification

Genomic DNA from PS and SS fungal mycelia of *C. gloeosporioides* was extracted by cell lysis method as previously described by Chi et al. (2009): In brief, 10–20mg of both fungal mycelia grown on PDA media for 2–3days were taken into Eppendorf tubes containing extraction buffer, and the fungal cells were pulverized with an electric grinder. Cell lysates were centrifuged at 5,000×*g* for 10min, and the supernatant was transferred into fresh Eppendorf tubes containing 300μl of 2-propanol. The lysate was mixed with 2-propanol by inverting the tubes several times for the DNA precipitation and then centrifuged at 12,000×*g* for 10min. This time the supernatant was discarded completely and washed with EtOH and allowed to evaporate. The pellet was mixed with 50μl 1× TE buffer (1M KCl, 100mM Tris-HCl, and 10mM EDTA) to dissolve the DNA. For amplification of multilocus sequence analysis (MLSA) of the actin (ACT), internal transcribed spacer (ITS) rDNA, and β-tubulin (Tub2), the genomic DNA was amplified in a thermocycler (MyGenie 96/Therma Block, Bioneer, Korea) using primer pairs ACT-521F/ACT-783R, ITS1/ITS4, and T1/T2, respectively (White et al., 1990). The conditions for polymerase chain reaction (PCR) amplification were 95°C for 5min, 35cycles of 94°C for 45s, 60°C for 30s, 72°C for 45s, and final 72°C for 5min. The product size was approximately 575bp, which was visible on a 1.2% agarose gel under UV light. The PCR product was purified using a PCR purification kit (Biofact, Biofactory, Korea) according to the manufacturer’s instructions. The PCR product was sequenced by Solgent services (Solgent Inc., Daejeon, Korea). The above primers were also used for this purpose. The sequence was compared with the reference species of fungal pathogens in a genomic database using the NCBI BLAST tool. Sequence alignment and phylogenetic tree construction were performed in MEGA 7.0 (Biodesign Institute, Tempe, Arizona, United States).

### Media Optimization for the Development of Fungal Mycelia

The PS and SS pathogens of *C. gloeosporioides* were inoculated on different media to identify the best medium for growth and sporulation. In this experiment, four different media, such as PDA, glucose-peptone agar (GPA, glucose 10g, peptone 2g, KH_2_PO_4_ 0.5g, MgSO_4_-7H_2_O 0.5g, agar 15g, Water 1L), carrot juice agar (CJA, carrot juice 125–750ml, Streptomycin 100mg, agar 15g, water 1L, pH 5.1–5.5), and malt extract agar (MEA, Difco), were used. Mycelial discs (5mm diameter) cut from the growing margins of the fresh fungal culture were placed at the center of each Petri plate. The diameter (mm) of the mycelial colonies was measured 5days after incubation at 25°C.

### Sporulation and Appressorium Formation Assay

Each of the 20 *C. gloeosporioides* isolates possessing PS and SS was inoculated onto PDA plates and incubated at 25°C for 15days in the dark under *in vitro* conditions. To each plate, 3ml SDW was added to obtain conidial suspensions and a hemocytometer was used to determine their concentration. For the percentage of germination assays, conidia suspensions at the concentration of 1.5×10^5^ conidia/ml were used for each isolate. Conidial suspensions (100μl) were spread onto PDA plates at a concentration of 1.5×10^5^ conidia/ml. After 24h of incubation at 25°C in darkness, 100 conidia from each replicate were evaluated under a light microscope (BX400; Olympus, Tokyo, Japan) for the determination of conidial germination (%), and the width and length of the conidia after germination were measured using a light microscope. Further, the appressorium formation from the conidia by primary hyphae, followed by germ tube formation, was observed 48h after incubation at 25°C.

### Assay for Testing Effects of Fruit Ripeness and Temperature on Virulence

The PS and SS isolates of *C. gloeosporioides* from the symptomatic tissues were tested for their virulence in three different states (ripe fruits, unripe fruits, and fruit cross section) by artificial inoculation. A 10μl conidial suspension (10^5^ conidia/ml) was placed on surface-sterilized apple fruits that had been wounded by piercing with a pin to a depth of 1–2mm. Inoculation of apple fruits with SDW served as a control. After inoculation, fruits were incubated at 25°C under a 12-h light/12-h dark cycle for different durations. Disease symptoms were evaluated 7days after inoculation by measuring the symptoms in diameter (mm). To determine the effect of various temperatures on the symptom development of PS and SS of *C. gloeosporioides*, the surface-sterilized apple fruits were artificially inoculated at a range of temperatures (15, 20, 25, 30, and 35°C) under the same light/dark conditions as described above. Disease symptoms were recorded 7days after inoculation by measuring the symptoms in diameter (mm). Each treatment consisted of five replicates (fruits).

### Microscopic Observation

For scanning electron microscopy (SEM) analysis, the tissues were prepared following the method of Ge and Guest (2011) with minor modifications. Samples were collected at 1, 2, 3, and 4days after inoculation. Apples inoculated with pathogenic conidial suspensions were incubated for different durations (1–4days). Small portions of the inoculated apple area were cut and dried for 30min at 45°C. The dried samples were directly subjected to a gold coating in an ion coater. The prepared specimens were observed using the SEM (Hitachi S-2500C, Jeol Co. Japan) at 10, 15, and 20kV.

### Effect of Ethephon Treatment on PS and SS of *Colletotrichum gloeosporioides*

To determine the effect of ethephon treatment for disease induction of PS and SS on apples, surface-sterilized mature apple fruits artificially inoculated with 10μl conidial suspensions (10^5^ conidia/ml) were treated with ethephon (10μM) that had been wounded by piercing with a pin to a depth of 1–2mm. Inoculation of apple fruits with conidial suspensions served as a control. After inoculation, fruits were incubated at 25°C under 12-h light/12-h dark cycle for different durations (1–7days). Disease symptoms of both PS and SS were evaluated for 7days after inoculation by measuring the symptom length in diameter (mm). Each treatment consisted of five replicates (fruits).

### Assay for Appressorium-Mediated Penetration Ability

To test the penetration ability of germinated conidia through the CM, a sheet of sterile CM (4×4cm) was placed on top of PDA plates. Then, 10μl spore suspensions (10^5^ conidia/ml) of PS or SS isolates were mixed either with water or ethephon (10μM) and then placed at the center of the CM. CM was removed after 1–3days and observed for the growth of PS and SS isolates 48h after incubation at 25°C. Each treatment contained three replicates, and each experiment was performed at least twice.

### *In vitro* Comparison of Penetration Hyphae of PS and SS of *Colletotrichum gloeosporioides*

Conidia germination and appressorium formation from PS and SS of *C. gloeosporioides* were tested on a cover glass surface in ethephon (10μM) following the method described previously (Flaishman and Kolattukudy, 1994; Kim et al., 1998). Briefly, conidia from cultures grown on PDA for 3–5days were harvested and washed twice with ice-cold SDW. Conidia (10^5^ conidia/ml) were suspended in ethephon (10μM), and 10μl of the suspension was dropped onto the cellophane membrane attached to the glass slide. Conidial germination and formation of appressorium and primary hyphae in PS and SS upon treatment with ethephon were assessed during different durations (4, 8, 16, and 20h) of incubation at 25°C. At least 50 measurements per structure were measured with a ProgRes speedXT ^core^3 Imager microscope using differential interference contrast illumination.

### RNA Isolation and Library Preparation for RNA-Seq

Total RNA was extracted from various fungal mycelia using TRIzol reagent (Invitrogen, Carlsbad, CA, The United States) and RNeasy® plant mine kits (QIAGEN, United States). Harvested mycelia of *C. gloeosporioides* were ground vigorously using a mortar and pestle in liquid nitrogen, and the mycelia powder was transferred into a DEPC-treated mortar and ground well with 2ml of TRIzol® reagent for 2min. This suspension was centrifuged at 14,000×*g* for 10min at 4°C, and the supernatant was transferred to an RNeasy mini spin column. Total RNA was extracted using RNeasy plant mini kits, according to the manufacturer’s instructions. RNA was dissolved in RNase-free water and converted to cDNA immediately or stored at −80°C. A total of 18 samples from the two lanes were used for sequencing reactions. RNA sequencing libraries were prepared using an Illumina TruSeq RNA sample prep kit (Illumina San Diego, CA), according to the manufacturer’s protocol. Sequencing was performed on an Illumina HiSeq2000 instrument using the reagents provided in the Illumina TruSeq PE Cluster Kit (Illumina San Diego, CA).

### Analysis of RNA-Seq Data

The genome sequence of *C. gloeosporioides* was obtained from the NCBI genome database.^1^ The accession code of the sequence was NC_014483.2. In this study, we analyzed 14,000 annotated genes of *C. gloeosporioides*. After sequencing, the enrichment mRNA raw reads were mapped onto the reference genome of E681 using the BWA program. Sequence alignment map (SAM) files generated by mapping were converted to BAM files in a binary format and then sorted by chromosomal coordinates using the SAMtools program available (Cock et al., 2015). Mapped reads per annotated gene were counted using the Bam2readcount. To analyze the gene expression levels of PS and SS of *C. gloeosporioides*, the relative transcript abundance was measured in reads per kilobase of exon per million mapped sequence reads (FPKM; Mortazavi et al., 2008). The log_2_ ratios of the FPKM values were used to identify the differentially expressed genes (DEGs). Subsequently, DEGs were identified using the DEGseq package in R language (Wang et al., 2010). To determine the differences in pathogenicity among the three isolates of *C. gloeosporioides*, several known pathogenicity genes corresponding to DEG were obtained from the NCBI GenBank (Supplementary Table S1). Among these, the isocitrate lyase (ICL) gene which is involved in the penetration process from the appressorium in plant pathogenic fungi, such as *Colletotrichum* and rice blast fungus, was unusually downregulated. Therefore, ICL is one of the key enzymes in the glyoxylate cycle.

### Gene Ontology Enrichment

Gene Ontology (GO) terms were downloaded from the VertiGO database of MicrosOnline.^2^ The significance of enrichment for total GO terms was calculated by Fisher’s exact test, and the obtained values of *p* were adjusted for multiple hypotheses testing by *q*-value (Storey and Tibshirani, 2003). For constructing pathway databases, we used a python code script and the “WGET” program of the LINUX operating system. The pathway database was downloaded from the Kyoto Encyclopedia of Genes and Genomes (KEGG).^3^

### Quantitative Real-Time PCR

To validate the transcription level from RNA-seq analysis, cDNA was generated using an iScript cDNA synthesis kit (Bio-Rad, Hercules, CA, United States) with a random hexamer. DNase-treated total RNA was used as a starting material to generate cDNA according to the manufacture’s protocol. Quantitative real-time PCR (qRT-PCR) was performed using a CFX Connect Real-Time PCR Detection System (Bio-Rad), with 18S rRNA used as a reference gene. For qRT-PCR, the reaction mixture contained 10ng cDNA, 5pmol each of forward and reverse primers, and SsoAdvanced SYBR Green Supermix (Bio-Rad). All primers used in this study were designed using the PrimerQuest algorithm on the Integrated DNA Technologies (IDT) website (Supplementary Table S2).^4^ Thermal cycling conditions were as follows: denaturation at 95°C for 3min for polymerase activation, followed by 40cycles at 95°C for 10s, and 60°C for 30s. After the last reaction cycle, melting curves were obtained through a temperature ramp from 65°C to 95°C, with 0.5°C/s increments, to exclude nonspecific products. Each PCR run included a “no template” sample, and all tests were performed in triplicate. The cycle threshold (*C*t) value relative to the control sample was considered for the calculation of ΔΔ*C*t (difference between Δ*C*t values calculated from the difference between the *C*t of the target and the reference gene) for the samples. The constitutively expressed 16S ribosomal RNA gene was used as a control for normalization (Yu et al., 2015).

### Growth of Fungal Pathogens on Minimal Media

The minimal medium consisted of 6gL^−1^ NaNO_3_, 0.52gL^−1^ KCl, 0.52gL^−1^ MgSO_4_·7H_2_O, 1.52gL^−1^ KH_2_PO_4_, 0.001% thiamine, and 0.1% trace elements supplemented with 10gL^−1^ glucose. The acetate medium contained 50mM sodium acetate and agar (1.5%) as a solidifying agent. The fungal mycelial disk (6mm in diameter) was taken with a sterile cork borer and inoculated onto freshly prepared solid media, followed by incubation for 10days at 25°C.

### Statistical Analysis

The data were subjected to analysis of variance (ANOVA) using SAS JMP software (SAS Institute, Cary, NC, 1995). Differences among treatment means were assessed using the least significant difference (LSD) test, and significance was established at *p*<0.05. All experiments were performed at least twice. For each experiment, the data were analyzed separately, and the results of one representative experiments are presented.

## Results

### Two Distinct Types of Anthracnose Disease Symptoms in Apple Orchards

To assess the occurrence of anthracnose disease in apples, a survey was conducted from 2013 to 2015 on apple orchards at eight different locations within Gyeongbuk Province in Korea (Figure 1A; Supplementary Figure S1). The typical disease symptoms on apple fruits were initially observed as small, slightly sunken, circular, light brown to dark brown lesions, and later became conspicuously sunken as they enlarged (Figure 1B; Supplementary Figure S1A). When the lesions grew to be 1–3cm in diameter, the formation of acervuli was found in concentric rings around the point of infection, resulting in the production of a large number of conidia on the surface of infected fruit. The spore masses appeared to be creamy and salmon-colored. In cross section, the lesions were V-shaped (Figure 1B, inset) and acervuli were found just beneath the cuticle of the fruit, which was ruptured by the growth of conidiophores.

Together with these typical anthracnose symptoms, we noticed that a significant portion of initial lesions (small and dark specks on apple fruits) remained small until the harvesting season (Figure 1C; Supplementary Figure S1B). Upon recognition of two distinct types of symptoms, which we designated as “progressive symptom” (PS) and “static symptom” (SS), disease incidence was monitored separately for PS and SS each year (Figure 1D). Except for Uiseong in 2013, the incidence of PS was higher in all regions and years than that of SS. However, the incidence of SS is comparable to that of PS in many regions. Considering that the high incidence of SS negatively affects the marketability of apples, it is pivotal to understand the etiology of the two distinct symptoms.

### *Colletotrichum gloeosporioides* as a Causal Agent of PS and SS Through Molecular Identification

**Figure 2:**
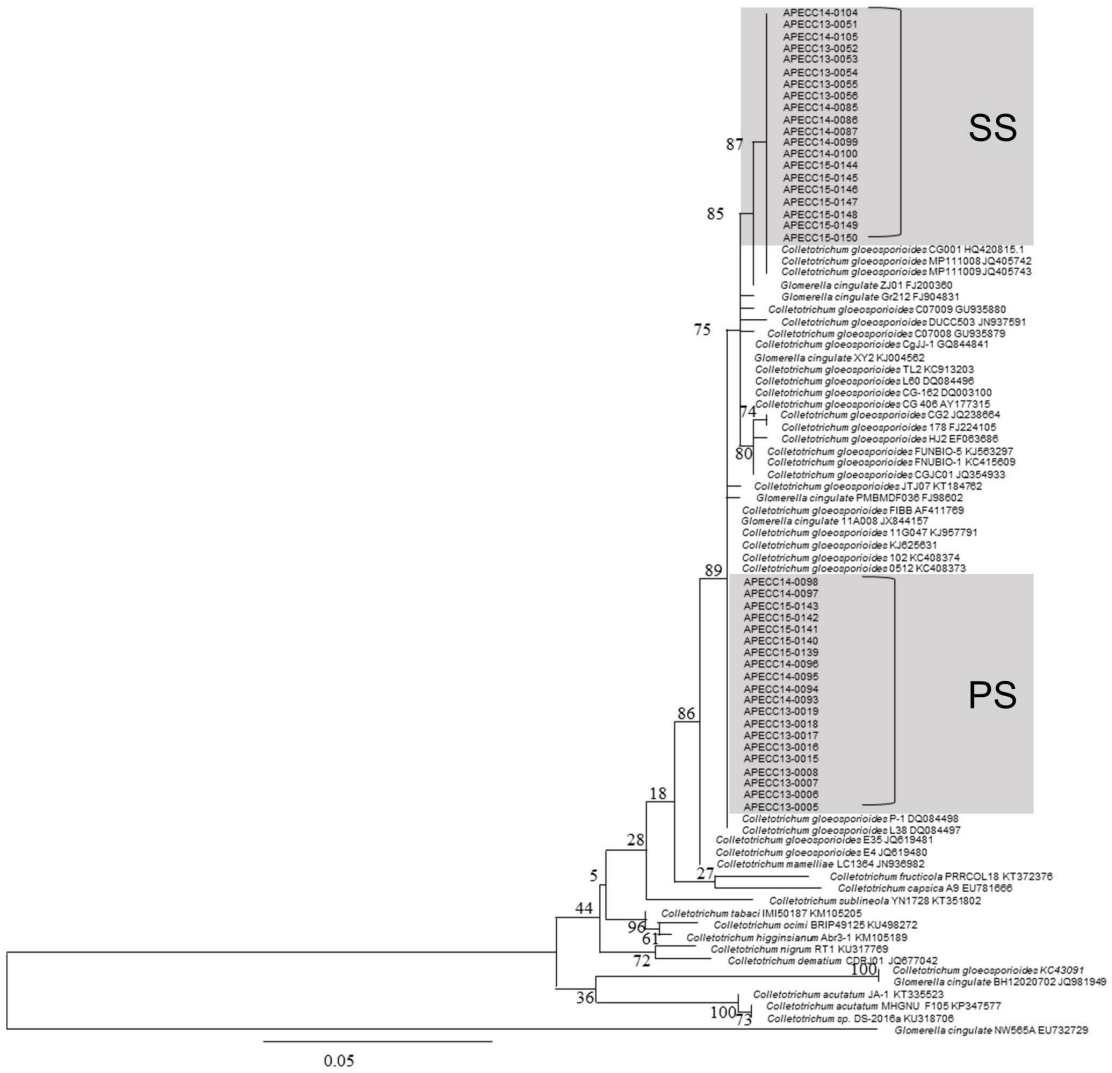
The phylogenetic tree of internal transcribed spacer (ITS) sequences of 40 isolates of *C. gloeosporioides* along with other isolates of *Colletotrichum* spp. from NCBI was inferred using the maximum likelihood method (MEGA 7.0). The percentage of replicate trees in which the associated taxa clustered together in the bootstrap test (100 replicates) is shown below the branches. The scale bar indicates 0.05 substitutions per nucleotide position.

During our survey, fungi were isolated from PS and SS lesions for identification and comparison. ITS sequencing of 139 isolates showed that they all belonged to *C. gloeosporioides*. Additional sequencing analysis of actin and β-tubulin for 18 randomly selected isolates corroborated the ITS sequencing results. To investigate whether there was a difference between PS and SS isolates at the genotype level, a phylogenetic tree was constructed using ITS sequences from our isolates that were representative of each lesion type in terms of sampling time, regions, and host varieties, together with sequences retrieved from GenBank (Figure 2).

Our phylogenetic analysis showed that PS and SS isolates are a part of the *C. gloeosporioides* species complex including *C. fructicola*, while they are more distantly related to species, such as *C. acutatum* and *C. tabaci*, which do not belong to the *C. gloeosporioides* species complex. Interestingly, PS and SS isolates tended to form closely related but distinct clades in our phylogenetic tree, although two PS isolates were found in SS clades, and vice versa. These results suggest that *C. gloeosporioides* is responsible for both PS and SS lesions in apples and that there are two subgroups within the *C. gloeosporioides* population in apple orchards of Gyeongbuk Province. The 40 fungal pathogenic isolates of *Colletotrichum* spp. Showed 98–100% similarity with the representative reference *C. gloeosporioides* isolates published in the NCBI GenBank database. Phylogenetic analysis of these isolates was clustered with reference *C. gloeosporioides* isolates (accession No. AF411769, DQ084497, and DQ084498) from NCBI GenBank with high bootstrap support (100%). Furthermore, 20 isolates and clustered with *C. gloeosporioides* (accession No. AY177315, KC913203; Figure 2).

### Comparison of PS and SS Isolates in Growth and Microscopic Observation

**Figure 3:**
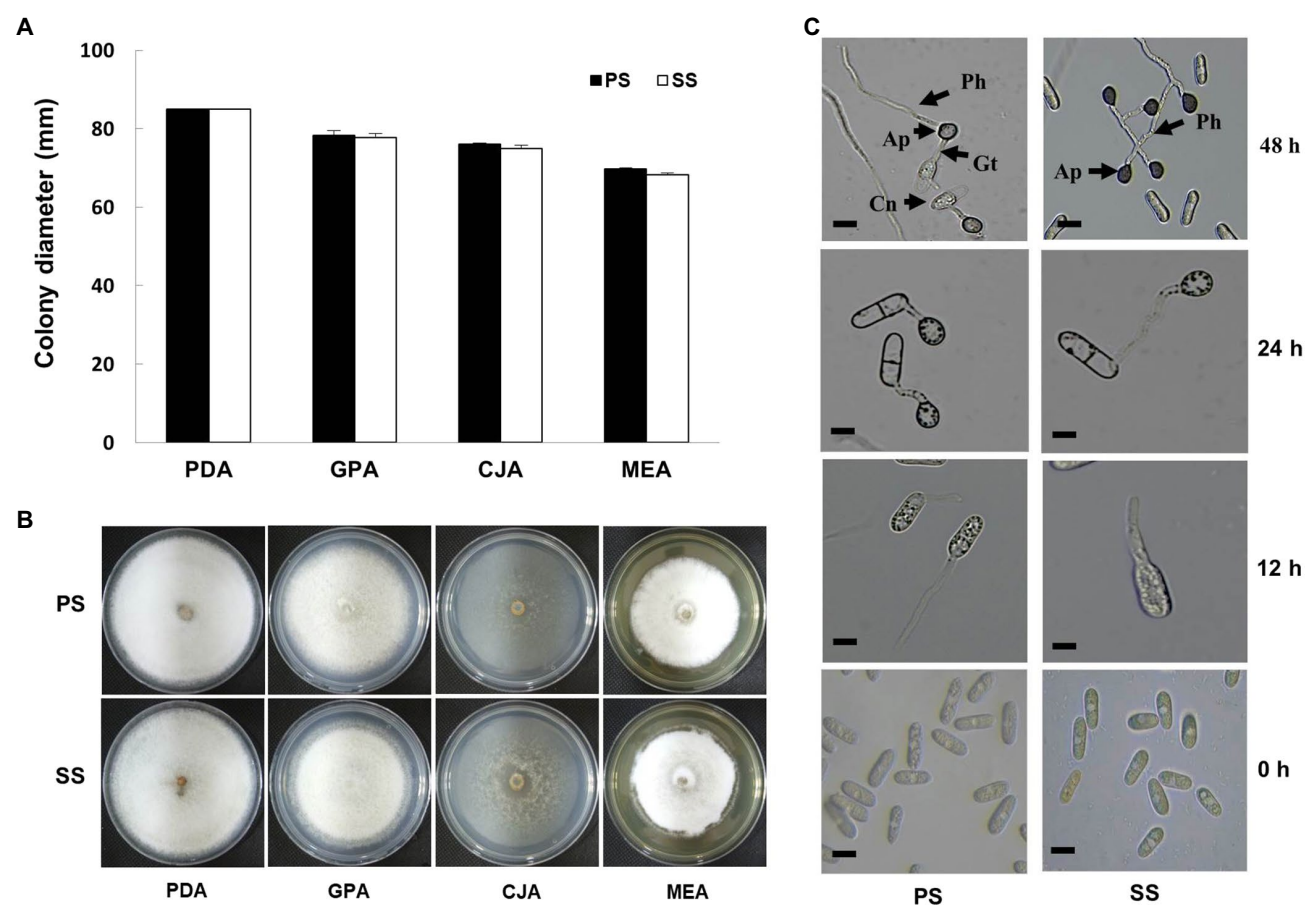
Effect of different media on mycelia growth and morphological characteristics of fungal pathogens. **(A)** Growth of mycelial colony (in diameter) of progressive (PS) and static (SS) fungal pathogens of *C. gloeosporioides* in different media was observed as similar in both cases. **(B)** Representative photographs of mycelial growth and characteristics of fungal mycelia of *C. gloeosporioides* as progressive and static on different media. Images were photographed 10days after incubation at 25°C. **(C)** Microscopic observation of appressoria formation and primary hyphae in water-treated conidia suspensions of PS and SS of *C. gloeosporioides* at different durations (0, 12, 24, and 48h). Bar=10μm. Ap, Appressorium; Cn, Conidium; Gt, Germ tube; Ph, primary hyphae.

Upon recognition of the two subgroups, each corresponding to PS and SS isolates, we set out to compare the phenotypes of these isolates. In addition, the growth rate of SS isolates, measured as colony diameter, was comparable to that of the PS isolates (Figure 3A). When grown on different media, colonies of both PS and SS isolates exhibited almost identical morphologies and pigmentation on different media (Figure 3B). Furthermore, microscopic observations revealed that the conidia of PS and SS germinated and developed appressoria on hydrophobic surfaces *in vitro* at different durations (Figure 3C). The conidial germination percentage ranged from 50 to 70 in both PS and SS conidia of *C. gloeosporioides* under a light microscope (Supplementary Table S3). Germ tubes were emerged from the PS conidium to develop an appressorium at 12h, while the germ tube was initiated by the conidium of the SS pathogen. The appressorium was formed from conidial germination by producing a shorter germ tube in PS than in SS after 24h of incubation. At this stage, conidia appeared in the two-celled stage. The primary hyphae were formed from the appressorium in the PS of the pathogen at 48h, while there was no formation of primary hyphae in the SS of the pathogen. The average size of appressoria was 6.7–14.3μm×5.3–8.7μm (length×width) in PS of the pathogen, while in SS the appressoria measured 6.5–12.0μm×5.0–6.9μm (length×width). Most appressoria were irregularly shaped, with dark brown, ovate, clavate, sometimes lobed, and thick walls in both types.

### Comparison of PS and SS Isolates for Their Virulence on Apples

**Figure 4:**
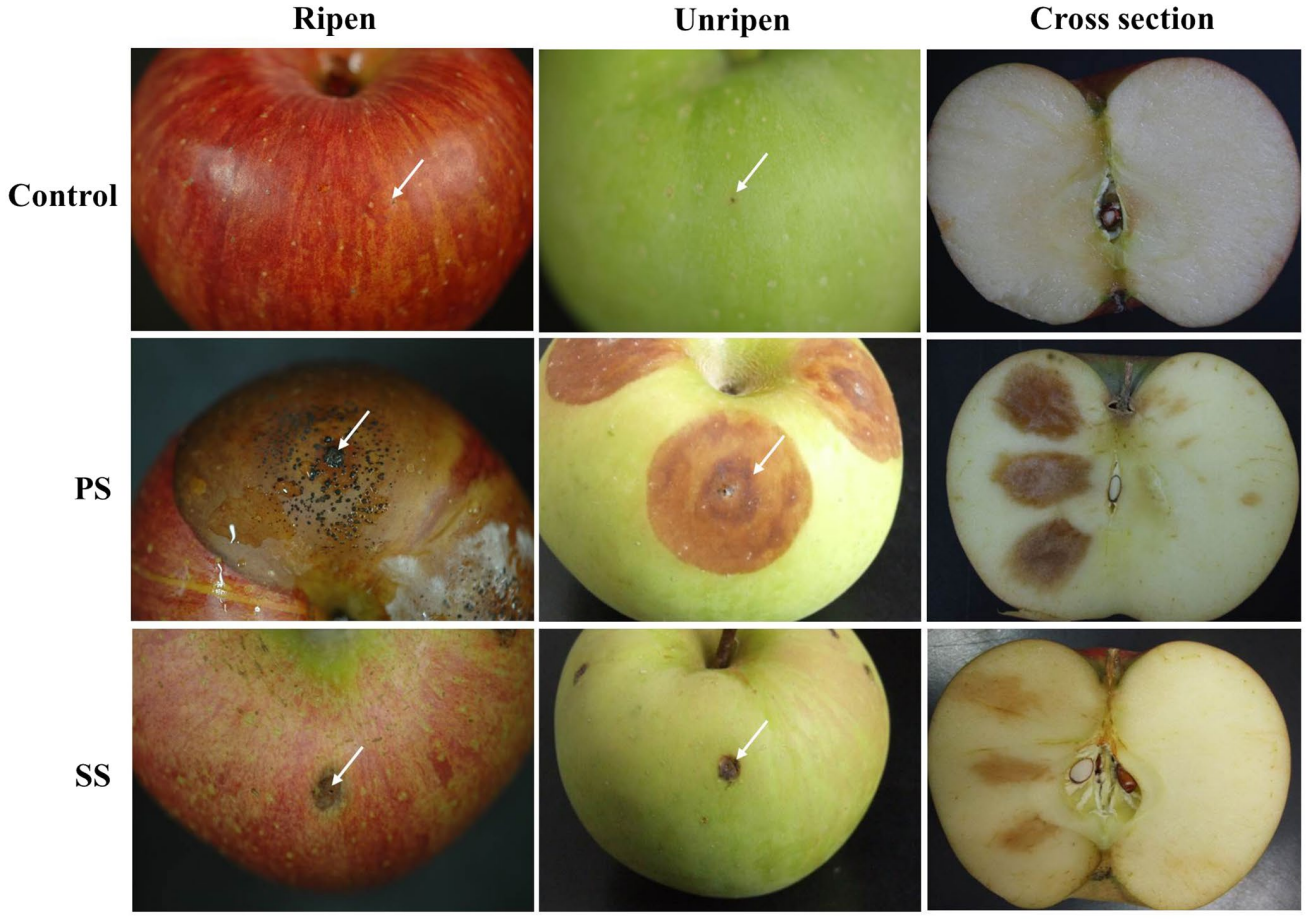
Virulence test of PS and SS on ripe and unripe apple fruits (cv. Fuji). Progressive symptoms (PS) of anthracnose appear as sunken lesions on ripe apples, with concentric circles of conidia. Static symptoms (SS) of anthracnose were not enlarged on ripe fruits after 7days of incubation at 25°C. Each treatment contains five replicates (fruits), and the experiment was performed twice.

To test the ability of the two isolates to cause anthracnose symptoms on apples, we artificially inoculated apples with PS and SS isolates. Although SS isolates were indistinguishable from PS isolates on different media, our pathogenicity assay repeatedly showed that SS isolates were not as capable of causing symptoms similar to ripe apples as PS isolates were under laboratory conditions (Figure 4). In contrast to PS isolates causing enlarging lesions with the formation of acervuli, SS isolates caused small lesions that did not grow large enough to develop acervuli.

Next, we investigated whether the virulence of PS and SS isolates could be altered by other factors such as the degree of apple ripeness and temperature. When unripe apples were used for pathogenicity assay, changes in lesion formation were observed in apples inoculated with PS, while essentially no difference was found for SS isolates between ripe and unripe fruits (Figure 4). Lesions formed by PS isolates on unripe apples were slightly smaller and light brown in color, compared to larger, dark brown lesions with acervuli at their center on ripening apples, suggesting that there is a link between ripeness and lesion development by PS isolates. In line with this result, inoculation with PS isolates following treatment of apple fruits with ethephon, a plant growth regulator helped fruits reach ripeness more quickly and enhanced disease development (Supplementary Figure S2). Unlike PS, ethephon had no effect on the development of SS lesions.

The effect of the temperature on lesion formation was examined in addition to ripeness. Our data showed that temperature can modulate lesion formation in PS isolates (Supplementary Figure S3). Low (15–20°C) and high temperatures (35°C) led to smaller lesion sizes than mid temperatures of 25–30°C in apples inoculated with PS isolates. However, the temperature had no effect on lesion development in SS isolates and our results strongly suggest that there is an intrinsic difference in virulence between PS and SS isolates, although environmental and host conditions can impact the course of lesion development.

### Sporulation, Germination, and Appressorium Formation in PS and SS Isolates

To identify what underlies the observed difference in virulence between PS and SS isolates, sporulation, conidial germination, and appressorium formation were examined. When spores were harvested from the culture plates and enumerated, we found that the PS and SS isolates produced a similar number of spores. In addition, spores of both isolates were able to germinate and form appressorium without any delay under laboratory conditions, suggesting that they are both capable of pathogenic development (Supplementary Figure S4).

Without apparent defects in asexual and pathogenic development, we then checked whether the appressoria of PS and SS isolates were functional by placing a drop of spore suspension on a transparent CM, which was placed on top of the media. If spores developed functional appressoria, they would penetrate the CM into the underlying media, leading to the formation of a fungal colony. We found that PS isolates showed colony formation on the media regardless of the presence of CM, while the SS isolate showed colony formation only on media without CM, suggesting that SS isolates are defective in appressorium-mediated penetration of the underlying substratum (Supplementary Figure S5).

### Effect of Ethephon Treatment on Conidial Germination and Appressorium Formation From PS and SS of *Colletotrichum gloeosporioides* Under *in vitro* Conditions

Ethephon-treated conidia of PS and SS on CM placed onto PDA plates responded similarly to water-treated conidia (Supplementary Figure S6A). However, ethephon-treated conidia from both isolates resting on the hard glass surface showed drastically enhanced germination and appressorium formation levels when compared to the water-treated conidia. These showed that the induction of appressorium formation varied in both PS and SS pathogens (Supplementary Figure S6B). Ethephon-treated conidia incubated on a hard surface at the required concentrations showed rapid germination at 4h in PS, while there was no germination in the SS pathogen at 4h in ethephon-treated conidia. Conidia of PS germinated and formed appressorium after 8h of incubation, but in the SS pathogen, the germ tube was only induced from conidium. Even at 8h, there was no formation of appressorium in SS, but many appressoria were observed in PS at 8h after ethephon treatment. After 20h, penetration hyphae were formed in PS, while no penetration hyphae were formed in SS; instead, the germ tube was elongated, resulting in delayed disease spread on apple fruits.

### Comparative RNA-Seq Analysis of PS and SS Isolates

**Figure 5:**
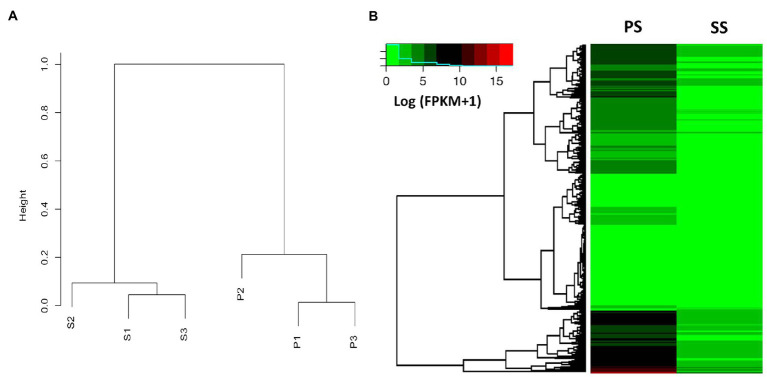
Dendrogram and heat map analysis between PS and SS of anthracnose caused by *C. gloeosporioides* on apples. **(A)** Dendrogram showing the expression values (FPKM) of each gene calculated by the Pearson correlation coefficient as a distance between the two samples and the distance between the replicates within each sample that is much closer compared to other samples. The variation within samples is much smaller than the variation between samples. **(B)** Genome-wide analysis of differentially expressed genes (DEGs) of PS and SS from apple anthracnose, using heat map analysis based on the Kyoto Encyclopedia of Genes and Genomes (KEGG) pathway mapper. Relative expression profiles of PS genes compared to those of the SS genes. Hierarchical clustering of genes based on the expression ratio ranks between PS and SS. As a result, the 3,353 genes were grouped into four clusters. The fold change is represented by color (see the color bar scale).

**Figure 6:**
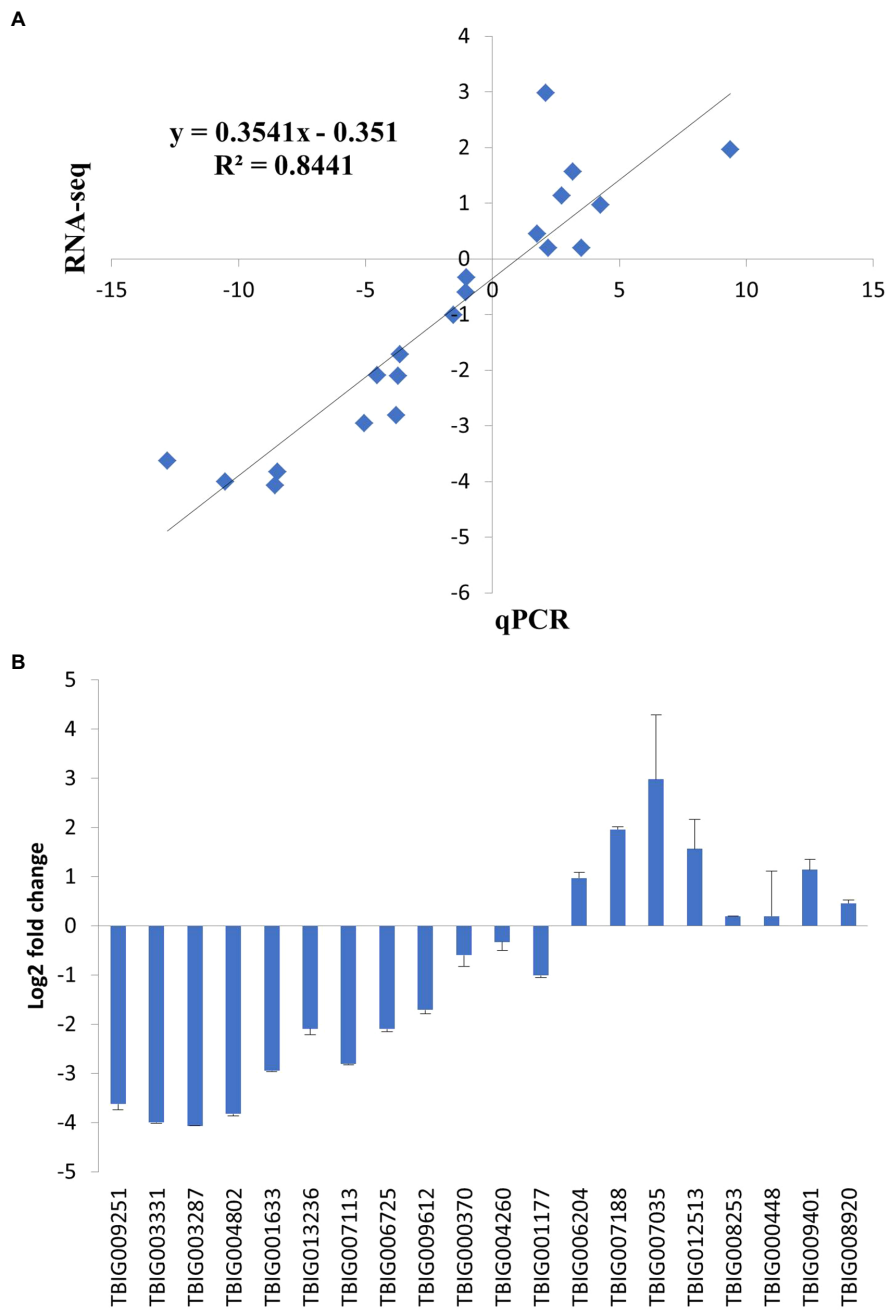
Validation of RNA-seq results using quantitative real-time PCR (qPCR). The relative expression levels of randomly selected genes were analyzed by qPCR to validate RNA-seq results. Each fold change was calculated by qPCR using the 2^−∆∆*C*t^ method, and the log_2_ ratio of the obtained values was compared with the log_2_ ratio of RNA-seq results, which were based on normalized *in vivo* FPKM to FPKM by DEGseq. **(A)** Correlation of fold change measured between RNA-seq (*x*-axis) and qPCR (*y*-axis). **(B)** Log_2_ fold change measured by qPCR. Error bars represent standard deviation.

To expand our observations, we compared the transcriptomes of PS and SS isolates using RNA-seq. Total RNA was extracted from fungal cultures grown on PDA media. For each isolate, three independent samples were obtained (in total, six samples) and subjected to RNA sequencing. The transcriptomes tended to be similar among the within-isolate samples, while those of PS and SS were quite distinct from one another (Figure 5A). Hierarchical clustering of genes was performed based on the expression ratio ranks of the two different anthracnose symptomatic pathogens, PS and SS, which *C. gloeosporioides*. As a result, 14,000 genes were grouped into clusters (Figure 5B). Analysis of PS and SS transcriptomes showed that approximately 31% of genes (*n*=3,993) were differentially expressed in the SS isolate, compared to the PS isolate, with statistically significant differences and a two-fold expression change cutoff. Among the DEGs, approximately 45.2% (*n*=1,804) and 54.8% (*n*=2,189) were upregulated and downregulated, respectively, in the SS isolate (Supplementary Tables S4 and S5). RNA-seq results were validated through the qRT-PCR analysis of genes (*n*=20) randomly chosen from DEGs and non-DEGs (Figure 6A). Following qPCR, the log_2_ fold changes in the relative gene expression of PS relative to SS samples were calculated, and the results were compared to RNA-seq data by scatter plotting. The log_2_ fold changes detected for each gene from RNA-seq analysis were similar to the results of qPCR, with a correlation coefficient (*R*^2^) as high as 0.84 (Figure 6B). Overall, similar patterns were found between RNA-seq and qRT-PCR (Pearson correlation coefficient=0.92), indicating that we accurately detected changes in gene expression between PS and SS.

### Altered Expression Profile of SS Isolates, Compared to PS Isolates

**Figure 7:**
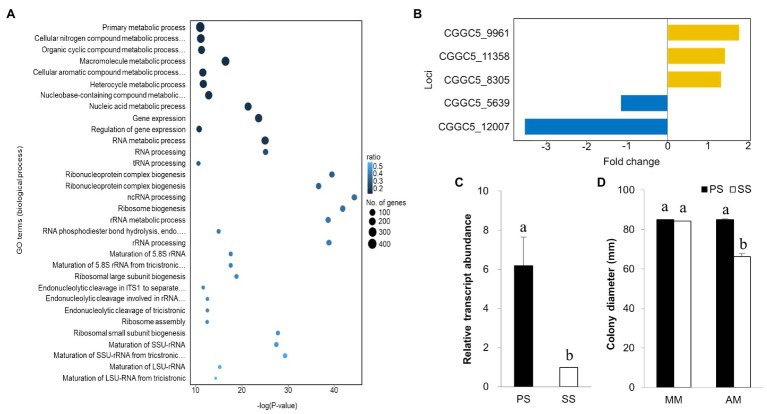
Gene ontology, expression of virulence gene, and expression of isocitrate lyase gene. **(A)** Gene ontology analysis for downregulated genes in both SS and PS in biological process based on functional categories. **(B)** Expression of the pathogenicity gene using RNA-seq analysis and comparison with other known virulence genes from NCBI. Isocitrate lyase gene expression in the PS pathogen at a greater level (upregulation) in comparison with SS strains. **(C)** The gene expression was greater in PS than in SS strain of *C. gloeosporioides*. **(D)** Mycelial growth of PS and SS of *C. gloeosporioides* in two different media [minimal medium (MM) and acetate medium (AM)]. SS was unable to utilize acetate as the sole carbon source for its growth. Bars with the same letters do not statistically differ from each other, according to the least significant difference (LSD) at *p*<0.05.

To gain insight into transcriptomic changes that might underlie differential virulence of PS and SS isolates, we performed gene ontology term analysis to quantify the enrichment of genes associated with biological processes. Our GO term analysis revealed interesting trends among DEGs: (i) DEGs were generally enriched with genes involved in a wide range of metabolic processes, (ii) upregulated genes in SS isolates showed enrichment of functions involved in the regulation of gene expression, RNA processing, and ribosome-related processes, whereas (iii) downregulated genes in the SS isolate displayed enrichment with genes implicated in response to oxidative stress, mitochondria-related processes, and transmembrane transport (Figure 7A; Supplementary Figure S7).

Although both upregulated and downregulated gene sets are enriched with functions in metabolic processes, the types of metabolic processes that are found in upregulated genes were different from those in downregulated genes. Upregulated genes are implicated in processes, such as primary metabolic processes, nitrogen compound metabolic processes, and nucleic acid metabolic processes (Supplementary Figure S7). In contrast, downregulated genes were found more frequently in processes, such as ribose phosphate metabolic process and purine ribonucleotide metabolic process, suggesting dramatic differences in the transcription of metabolism-related genes between PS and SS isolates.

To determine why the SS isolate is less virulent than PS, we used a list of known pathogenicity genes in *Colletotrichum* species and checked their expression in our data set. Among 36 known pathogenicity genes, 23 had corresponding orthologs in the genome of *C. gloeosporioides*. Of those, we found five genes were differentially expressed between the PS and SS isolates. Genes encoding putative cAMP-dependent protein kinase subunit, ammonium transporter Mep2, and nitrogen response regulator were upregulated, while laccase and ICL genes were downregulated in SS isolate (Figure 7B). ICL, one of the principal enzymes in the glyoxylate cycle, has been reported as a pathogenic gene in many pathogens (Dunn et al., 2009). The qRT-PCR analysis confirmed significant downregulation of the ICL gene in the SS isolate compared to PS (Figure 7C). Since the glyoxylate cycle is required for the utilization of fatty acids and/or lipids through the conversion of acetyl-CoA to succinate for the synthesis of carbohydrates, we hypothesized that SS isolates should be compromised in their ability to utilize carbon sources, such as ethanol, acetate, or oleic acid. Therefore, we tested the ability of PS and SS isolates to grow on minimal media (MM) with acetate as the sole carbon source (Figure 7D). On basic MM, the growth of the SS isolate was comparable to that of the PS isolate. However, the SS isolate showed a significant reduction in radial growth when acetate was the sole carbon source in the MM, suggesting that the SS isolate was not able to use acetate as efficiently as the PS isolate. This may explain the delay in penetration hyphae formation from the appressorium in the SS pathogen.

## Discussion

Symptoms or lesions are visible changes that occur in host plants in response to pathogen infection. The presence of different lesion types, caused by a particular pathogen on a single host plant tissue, is well-known; however, they are usually attributed to differences in microenvironments (Kruger et al., 2002; Rosenberg et al., 2007; Surico, 2013). In this study, we investigated two distinct types of anthracnose disease lesions and found on apple fruits, which were designated as PS and SS. A 3-year survey of apple orchards showed that small specks, which do not expand over time (static symptoms), are almost as prevalent as typical enlarging anthracnose symptoms (progressive symptoms). Isolation of fungal pathogens from the lesions and ITS sequencing indicated that both PS and SS isolates belonged to the anthracnose pathogen *C. gloeosporioides*. Species identification was corroborated by the sequencing of the actin and tubulin genes. The *C. gloeosporioides* species complex comprises more than 20 subspecies, based on a strongly supported clade within the *Colletotrichum* ITS gene tree (Weir et al., 2012; Yokosawa et al., 2017). Although PS and SS isolates formed different clades in our phylogenetic analysis, they appeared to be *C. gloeosporioides sensu stricto*, with branch lengths between the clades too short to support separation at the subspecies level.

Our pathogenicity assay using PS and SS isolates recapitulated progressive and static lesions, respectively. Furthermore, changes in temperature and degree of fruit ripeness did not affect the outcome of pathogen infection, either by PS or by SS. These results indicate that differences in virulence of PS and SS isolates appear to be intrinsic to each isolate, rather than being determined by environmental factors. When the sporulation, germination, and appressorium formation were monitored, few differences were detected between the two isolates. However, we found that SS isolate was not able to penetrate the cellophane membrane, suggesting some defects in the appressorium-mediated infection process. However, such defects do not fully account for the formation of small specks on apple fruits, since there is no infection at all if the SS isolate is completely incapable of penetration.

To explain why the SS isolate developed static symptoms, we performed a comparative analysis of the transcriptomes. Our RNA-seq experiments showed that approximately one-third of the genes were differentially expressed between the PS and SS isolates. Detailed analysis of DEGs revealed that differences in transcript abundance were found primarily in genes involved in diverse metabolic processes. These results strongly suggest that distinct transcriptional programs leading to differences in metabolic capacity underlie differences in symptom development. In line with this, we found that a gene encoding putative isocitrate lyase (*CgICL1*) was highly downregulated in the SS isolate, compared to the PS isolate. ICL is a key enzyme in the glyoxylate cycle that catalyzes the cleavage of isocitrate to succinate and glyoxylate. The glyoxylate cycle provides a means for cells to convert two acetyl-CoA units, generated by various catabolic processes into C_4_ units (succinate), which can be used to replenish the TCA cycle or to serve as precursors for the biosynthesis of amino acids and carbohydrates (Ensign, 2006; Kunzea et al., 2006; Schneider et al., 2012). In many plant fungal pathogens, of plants including *Magnaporthe oryzae* and *Colletotrichum lagenarium*, it has been demonstrated that genes encoding ICL are required for full virulence (Xue et al., 2002; Wang et al., 2003; Asakura et al., 2006). Taken together, our data suggest that genome-wide changes in the transcription pattern of metabolic genes, including *CgICL1*, between two isolates of *C. gloeosporioides* might be responsible for the observed difference in disease symptom development.

Although we claimed, based on sequencing of multiple markers, that both PS and SS isolates belong to *C. gloeosporioides*, it is not clear how identical they are at the genome level. Sequencing and comparative analysis of whole genomes may yield some insights into this knowledge gap. It should also be noted that epigenetic factors, such as DNA methylation and/or histone modifications, might contribute to differences between PS and SS isolates. However, the fact that they all are *C. gloeosporioides* raises the question of why and how the expression patterns of metabolic genes have diverged so much between PS and SS. During host-pathogen interactions, efficient nutrition of pathogens is a prerequisite for successful colonization of host plants (Casadevall and Pirofski, 1999; Divon and Fluhr, 2007; Selin et al., 2016; Araz et al., 2017). In fungal pathogens of plants, the ability to adapt to exploit nutrients from host cells must have developed within the context of host physiology and metabolism (Baarlen et al., 2007; Bellincampi et al., 2014; Zeilinger et al., 2016). Therefore, it may be that the SS isolate is adapting to different host plants other than apples. One observation made during our survey on apple orchards, which may be relevant to the origin of the SS isolate, is that false acacia trees (*Robinia pseudoacacia*) are commonly found around apple orchards and their leaves are often heavily infected with *C. gloeosporioides* (data not shown). Thus, one possibility is that the static symptoms of apple anthracnose are caused by spores of *C. gloeosporioides* dispersed from the false acacia trees. Further studies should focus on clarifying the relationship among PS, SS, and acacia isolates.

In summary, we have shown that different symptoms in apple fruit can be caused by two sub-populations of *C. gloeosporioides*, which are distinct in their transcriptional program. It is not clear whether sub-populations are a general phenomenon for fungal pathogens in plants. The reasons for this degree of transcriptional divergence within the same species remain enigmatic, but our study supports that seemingly identical populations in the field may consist of different sub-populations at the transcriptome level. This finding may have ramifications for molecular studies using a single, representative isolate of the pathogen, as well as ecological studies aimed at understanding the population dynamics of pathogens.

## Data Availability Statement

The datasets presented in this study can be found in online repositories. The names of the repository/repositories and accession number(s) can be found at: https://www.ncbi.nlm.nih.gov/genbank/, NC_014483.2.

## Author Contributions

WC and YK designed the experimental setup and performed the laboratory experiments. YL performed the RNA sequencing experiment. HK was involved in the field survey. KB and JJ analyzed the data and wrote the manuscript. YJ supervised the project. All authors contributed to the article and approved the submitted version.

## Funding

This work was supported by the Korea Institute of Planning and Evaluation for Technology in Food, Agriculture, Forestry, and Fisheries (IPET) through the Strategic Initiative for Microbiomes in Agriculture and Food, funded by the Ministry of Agriculture, Food and Rural Affairs (MAFRA; Grant No. 918009-4).

## Conflict of Interest

The authors declare that the research was conducted in the absence of any commercial or financial relationships that could be construed as a potential conflict of interest.

## Publisher’s Note

All claims expressed in this article are solely those of the authors and do not necessarily represent those of their affiliated organizations, or those of the publisher, the editors and the reviewers. Any product that may be evaluated in this article, or claim that may be made by its manufacturer, is not guaranteed or endorsed by the publisher.

## Footnotes

^1^ http://www.ncbi.nlm.nih.gov/genome/

^2^ http://www.microbesonline.org

^3^ http://www.genome.jp/kegg/

^4^ http://www.idtdan.com/Scitools/Applications/Primerquest/
