# Supplementary material for: Distinct Transcriptional Programs Underlie Differences in Virulence of Isolates on Host Plants in a Fungal Pathogen, Colletotrichum gloeosporioides
Source: Front Microbiol. 2021 Nov 8;12:743776. doi: 10.3389/fmicb.2021.743776 (PMC8630545; doi:10.3389/fmicb.2021.743776)
Supplement: Supplementary file 1 [file Data_Sheet_1.docx]

**Supporting Information**

**Distinct transcriptional programs underlie differences in virulence of isolates on host plants in a fungal pathogen, *Colletotrichum gloeosporioides***

Wonsu Cheon^1, 2^, Young Soo Kim^1, 3^, Kotnala Balaraju^4^, Younmi Lee^1, 4^, Hyeok Tae Kwon^1^, Junhyeon Jeon^5*^, Yongho Jeon^1*^

^1^Department of Plant Medicals, Andong National University, Andong 36729, Korea

^2^Microbial Research Department, Nakdonggang National Institute of Biological Resources, Sangju 37242, Korea

^3^Central Research Institute, Kyung Nong Co. Gyeongju 38175, Korea

^4^Agricultural Science & Technology Research Institute, Andong National University, Andong 36729, Korea

^5^Department of Biotechnology, Yeungnam University, Gyeongsan, Gyeongbuk 38541, Korea

Co-Corresponding authors:

**Prof. Yongho Jeon,**

E-mail: [yongbac@andong.ac.kr](mailto:yongbac@andong.ac.kr)

Tel: +82-54-820-5507

Fax: +82-54-820-6320

**Prof. Junhyeon Jeon,**

E-mail: [jjeon@yu.ac.kr](mailto:jjeon@yu.ac.kr)

Tel: +82-53-810-3030

Fax: +82-53-810-4769

**Supplementary Tables**

**Table S1.** Known pathogenicity genes by NCBI

| **Gene name** | **Locus ID** | **Species** | **References** |
| --- | --- | --- | --- |
| DN3 | GCU94180 | *Colletotrichum gloeosporioides* | Sally-Anne *et al.* |
| Cgl-SLT | JQ322774 | *Colletotrichum gloeosporioides* | Yong *et al.* |
| CgPKAC | DQ812968 | *Colletotrichum gloeosporioides* | Priyatno *et al.* |
| laccase gene | AY853696 | *Colletotrichum gloeosporioides* | Guetsky *et al.* |
| PELB (pectate lyase B gene) | AF052632 | *Colletotrichum gloeosporioides* | Yakoby *et al.* |
| STUA  (APSES transcription factor gene) | EF408244 | *Colletotrichum gloeosporioides* | Zhang *et al.* |
| ICL1 (isocitrate lyase) | AB246699 | *Colletotrichum lagenaria* | Makoto *et al.* |
| LAC1 (laccase) | AB055709 | *Colletotrichum lagenaria* | Gento *et al.* |
| kap1 (putative importin) | FM201303 | *Colletotrichum higginsianum* | Huser *et al.* |
| ATP citrate lyase | KM818515 | *Colletotrichum truncatum* | Jonathan *et al.* |

**Table S2.** Primer sequences used for quantitative RT-PCR.

| **Name** | **Forward Primer (5’ - 3’** | **Reverse Primer (5’- 3’)** | **Description of target gene** |
| --- | --- | --- | --- |
| TBIG009251 | AACTGACGCAGCCTATCT | CTACTTACCCAGTCACCTTCT | hypothetical protein |
| TBIG003331 | CACAACAAGCAAACCAACC | TTGGAAGAGAGTGCCAGA | conidiation-specific expression protein |
| TBIG003287 | GACGAGGGAGACATTGAGTA | AATTCGTTCCGCCCAAG | tubulin alpha chain |
| TBIG004802 | GCAGGACGATTGATGCTAATA | GTGTCCCATCACTTTCCTTAC | glutathione s-transferase |
| TBIG001633 | GGGCAACAAGAAGCAAGA | GCTGACACCGATTGACTTT | NADPH-dependent alpha-keto amide |
| TBIG013236 | AACTTCATATCCCGCTTCAC | AGGGCGATGCAGATGTA | fungal specific transcription factor domain-containing protein |
| TBIG007113 | CCAACGATGAGACAGAACAA | ATACCTTCCATTGCCCAAATA | methyltransferase domain-containing protein |
| TBIG006725 | GGCGATCAAGCAGTACAA | GGTAGTCGTTGAACCATAGG | cellulose-binding gdsl lipase |
| TBIG009612 | CGCTCTTGCTCTCATCATATC | GTAGGAGTCAGTTGGGACTT | maltose high-affinity maltose transporter |
| TBIG000370 | GTTCTGAGTCCCAGTCCTA | CCGCATCTCAGCCATAAA | SAM dependent methyltransferase, putative |
| TBIG004260 | TACTCTGACATCCCTCTTACC | AAACCTGGACCTCTTCCT | monocarboxylate permease-like protein |
| TBIG001177 | GCCCGTATGCACGATATG | CGCTACTTCTTCCGACTTTC | ATP-dependent RNA helicase dbp6 |
| TBIG006204 | AGCGAACGACGAGATAGA | GACAGTCAGTGCTGGTTATT | oligopeptide transporter |
| TBIG007188 | ACCGCCTACCTTACGATTA | TTCCAGGGCTCTGAATTTG | fungal specific transcription factor |
| TBIG007035 | GCAACTCCGACCTCTACTA | CGAGTTCGTGGAGCTTATTC | isoamyl alcohol oxidase |
| TBIG012513 | CGCGAATTCTACAGCAAGA | GATAAAGGCCTGGAGGAAAC | potassium ion channel yvc1 |
| TBIG008253 | GAAATGGGCGGGAAGATAC | CCTCGGACCAGATTTGATAAC | acetylhydrolase |
| TBIG000448 | CTACCTGTTCTGGCCTTTG | GAGAGGGCTTCTCAACATTAG | zinc metalloproteinase |
| TBIG009401 | GTTGAGTTCCTTCTGGTCTTC | CGTTAGTTCCTGCATGGTTTA | oxalate decarboxylase |
| TBIG008920 | GAGAAGGTTGGCGGTATTC | CTGGACGGCAATGTCAAA | integral membrane protein |
| TBIG011766 | GACTTGCGTCGTCCAAATA | GTTTCCTGTCGTGGAAGAG | isocitrate lyase |

**Table S3.** Mean values and standard errors for conidial germination and conidial measurements from various isolates of *Colletotrichum gloeosporioides*

|  | **Conidia germination**  **(%)^x^** | **Appressoria formation**  **(%)^x^** | **Conidia measurements** | |
| --- | --- | --- | --- | --- |
| **Isolate** | **24h** | **48h** | **Width (µm)** | **Length (µm)** |
| **Progressive** |  |  |  |  |
| APECC13-0005 | 62.6 ± 1.11 abc | 54.1 ± 3.09 abcde | 6.37 ± 0.13 a | 16.34 ± 0.06 abc |
| APECC13-0006 | 73.1 ± 1.08 a | 59.8 ± 1.8 a | 5.97 ± 0.33 abc | 15.30 ± 0.60 abcde |
| APECC13-0007 | 68.1 ± 7.20 ab | 52.9 ± 4.10 abcdefg | 5.62 ± 1.27 abcd | 15.54 ± 0.79 abcde |
| APECC13-0008 | 66.3 ± 0.91 abc | 50.3 ± 5.0 abcdefghi | 5.71 ± 0.42 abcd | 15.68 ± 0.72 abcde |
| APECC13-0015 | 68.9 ± 3.15 ab | 55.9 ± 2.8 abcdef | 5.38 ± 0.61 abcde | 15.91 ± 0.71 abcde |
| APECC13-0016 | 68.0 ± 6.53 ab | 54.8 ± 2.8 abcdef | 5.01 ± 0.44 abcdef | 15.76 ± 0.76 abcde |
| APECC13-0017 | 59.2 ± 8.62 bc | 53.0 ± 1.1 abcdefg | 4.88 ± 0.47 cdefgh | 16.54 ± 0.57 a |
| APECC13-0018 | 56.6 ± 4.59 bc | 57.2 ± 1.4 abcd | 5.57 ± 0.63 abcd | 16.47 ± 0.40 ab |
| APECC13-0019 | 55.5 ± 5.98 bc | 55.2 ± 0.4 abcdef | 5.70 ± 0.55 abcd | 14.16 ± 0.14 cde |
| APECC14-0093 | 62.3 ± 3.73 abc | 52.0 ± 2.0 abcdefgh | 5.61 ± 0.40 abcd | 15.76 ± 0.70 abcde |
| APECC14-0094 | 63.3 ± 3.87 abc | 48.4 ± 10.1 bcdefghij | 6.30 ± 0.09 ab | 15.09 ± 0.79 abcde |
| APECC14-0095 | 63.3 ± 9.25 abc | 58.0 ± 2.0 abc | 5.32 ± 0.52 abcde | 16.22 ± 0.46 abcd |
| APECC14-0096 | 69.2 ± 1.80 ab | 56.2 ± 3.67 abcde | 5.40 ± 0.66 abcde | 16.31 ± 0.43 abc |
| APECC14-0097 | 65.5 ± 9.28 abc | 48.5 ± 5.2 bcdefghij | 4.79 ± 0.79 cdefgh | 15.90 ± 0.63 abcde |
| APECC14-0098 | 68.8 ± 5.43 bc | 49.9 ± 5.6 abcdefghi | 5.66 ± 0.58 abcd | 14.96 ± 0.97 abcde |
| APECC15-0139 | 52.8 ± 7.12 c | 49.4 ± 3.2 bcdefghi | 5.42 ± 0.41 abcde | 15.76 ± 0.84 abcde |
| APECC15-0140 | 61.3 ± 1.41 abc | 58.0 ± 2.9 abc | 6.00 ± 0.42 abc | 14.52 ± 1.63 abcde |
| APECC15-0141 | 62.7 ± 9.39 abc | 55.7 ± 3.0 abcdef | 4.61 ± 0.10 cdefghi | 14.26 ± 1.73 bcde |
| APECC15-0142 | 63.0 ± 1.70 abc | 45.9 ± 1.4 fghijk | 4.58 ± 0.08 cdefghi | 14.16 ± 1.94 cde |
| APECC15-0143 | 61.6 ± 1.78 abc | 58.6 ± 0.3 ab | 4.88 ± 0.27 bcdefg | 14.55 ± 1.58 abcde |
| **Static** |  |  |  |  |
| APECC13-0051 | 60.3 ± 1.82 abc | 48.1 ± 2.3 cdefghij | 5.09 ± 0.57 abcdef | 14.24 ± 0.78 bcde |
| APECC13-0052 | 58.3 ± 2.71 bc | 43.4 ± 1.2 ghijk | 5.15 ± 0.48 abcdef | 14.12 ± 0.82 cde |
| APECC13-0053 | 62.3 ± 1.41 abc | 42.3 ± 1.9 hijk | 5.16 ± 0.46 abcdef | 14.76 ± 0.13 abcde |
| APECC13-0054 | 65.5 ± 3.49 abc | 48.8 ± 1.0 bcdefghij | 4.35 ± 0.13 defghi | 15.43 ± 1.04 abcde |
| APECC13-0055 | 69.4 ± 3.33 ab | 43.0 ± 3.4 ghijk | 4.69 ± 0.29 cdefghi | 14.67 ± 0.35 abcde |
| APECC13-0056 | 64.0 ± 2.88 abc | 40.4 ± 2.9 ijk | 4.89 ± 0.41 bcdefg | 15.26 ± 0.11 abcde |
| APECC14-0085 | 56.1 ± 1.95 bc | 45.9 ± 2.0 fghijk | 3.33 ± 0.19 i | 14.24 ± 0.14 bcde |
| APECC14-0086 | 66.9 ± 3.18 ab | 46.9 ± 4.6 efghij | 5.22 ± 0.42 abcde | 14.16 ± 0.38 cde |
| APECC14-0087 | 62.9 ± 1.65 abc | 43.1 ± 6.9 ghijk | 4.89 ± 0.41 bcdefg | 14.03 ± 0.25 de |
| APECC14-0097 | 64.8 ± 2.28 abc | 40.4 ± 3.1 ijk | 3.56 ± 0.99 ghi | 11.81 ± 0.46 f |
| APECC14-0098 | 62.9 ± 3.15 abc | 38.8 ± 5.9 jk | 3.47 ± 0.69 hi | 13.76 ± 0.46 e |
| APECC14-0099 | 63.8 ± 2.36 abc | 47.4 ± 1.8 defghij | 3.36 ± 0.14 i | 14.26 ± 0.37 bcde |
| APECC14-0100 | 66.1 ± 3.05 abc | 41.7 ± 4.3 hijk | 4.13 ± 0.41 efghi | 13.88 ± 0.09 e |
| APECC15-0144 | 64.3 ± 4.24 abc | 36.8 ± 6.8 k | 3.77 ± 0.45 fghi | 14.48 ± 0.78 abcde |
| APECC15-0145 | 56.7 ± 1.06 bc | 46.7 ± 1.1 efghijk | 3.80 ± 0.41 fghi | 14.45 ± 0.80 abcde |
| APECC15-0146 | 63.1 ± 0.89 abc | 43.9 ± 1.6 ghijk | 4.75 ± 0.37 cdefgh | 14.67 ± 0.66 abcde |
| APECC15-0147 | 67.5 ± 2.05 ab | 44.2 ± 2.5 ghijk | 5.01 ± 0.70 abcdef | 13.90 ± 0.53 e |
| APECC15-0148 | 66.1 ± 2.79 abc | 42.1 ± 1.0 hijk | 4.61 ± 0.20 cdefghi | 13.97 ± 0.63 de |
| APECC15-0149 | 66.8 ± 7.37 ab | 45.7 ± 2.3 fghijk | 5.28 ± 0.18 abcde | 15.87 ± 0.71 abcde |
| APECC15-0150 | 64.7 ± 0.39 abc | 44.0 ± 1.0 ghijk | 5.52 ± 0.02 abcde | 16.70 ± 0.28 a |

**^x^**Mean values in the columns followed by the same letters are not significantly different, according to the Duncan’s multiple range test at *P* = 0.05.

**Table S4.** List of up-regulated genes (carbohydrate metabolic process) in the PS under SS

| **Gene** | **Description** | **Log_2_ fold chang**  **(SS/PS)** |
| --- | --- | --- |
| TBIG008303 | glycoside hydrolase family 16 protein | -10.6 |
| TBIG008296 | glycoside hydrolase family 79 protein | -7.98 |
| TBIG012050 | cell wall glucanosyltransferase mwg1 | -6.92 |
| TBIG002868 | d-xylulose 5-phosphate d-fructose 6-phosphate | -6.16 |
| TBIG004748 | mannose-6-phosphate class i | -4.11 |
| TBIG013731 | glycoside hydrolase family 43 | -4.01 |
| TBIG004702 | xylanase 3 | -3.84 |
| TBIG008609 | glycosyl hydrolase family | -3.72 |
| TBIG004946 | glycoside hydrolase family 27 protein | -3.52 |
| TBIG014266 | cell wall glucanase | -3.51 |
| TBIG003402 | chitinase 3 | -3.37 |
| TBIG001485 | glycoside hydrolase | -3.27 |
| TBIG013604 | glucan-beta-glucosidase | -3.14 |
| TBIG005198 | alpha-glucosidase | -3.05 |
| TBIG002668 | beta-galactosidase, putative | -2.76 |
| TBIG014231 | beta-glucosidase g | -2.36 |
| TBIG004984 | beta-glucosidase 1 precursor | -2.31 |
| TBIG010971 | glycosyl hydrolase family 2 | -2.24 |
| TBIG004199 | glycoside hydrolase family 3 domain protein | -2.19 |
| TBIG008513 | glycoside hydrolase family 16 | -2.14 |
| TBIG001918 | beta-glucosidase | -2.06 |

| **Gene** | **Description** | **Log_2_ fold chang**  **(SS/PS)** |
| --- | --- | --- |
| TBIG006465 | aconitate hydratase | -3.88126 |
| TBIG011766 | isocitrate lyase | -3.52838 |
| TBIG009393 | succinyl-synthetase subunit | -3.33279 |
| TBIG001863 | malate synthase | -2.6845 |
| TBIG015651 | succinate:fumarate antiporter | -2.67681 |
| TBIG010224 | malate l-lactate | -2.02388 |
| TBIG006730 | alpha-ketoglutarate dependent xanthine dioxygenase | -1.52442 |
| TBIG012053 | alpha-ketoglutarate-dependent sulfonate dioxygenase | -1.50122 |
| TBIG013649 | fumarate reductase | -1.43427 |
| TBIG009449 | citrate lyase beta | -1.28779 |
| TBIG003170 | isocitrate dehydrogenase | -1.25634 |
| TBIG012052 | alpha-ketoglutarate-dependent sulfonate dioxygenase | -1.18948 |
| TBIG002558 | citrate synthase i | -1.14961 |
| TBIG015210 | alpha-ketoglutarate dependent xanthine dioxygenase | -1.13093 |
| TBIG015209 | alpha-ketoglutarate dependent xanthine dioxygenase | -1.02857 |
| TBIG010550 | succinyl-synthetase beta | 1.442152 |
| TBIG008350 | alpha-ketoglutarate-dependent sulfonate dioxygenase | 1.617794 |

**Table S5.** List of up- and down-regulated genes (glyoxylate cycle) in the PS under SS.

**Supplementary Figures**


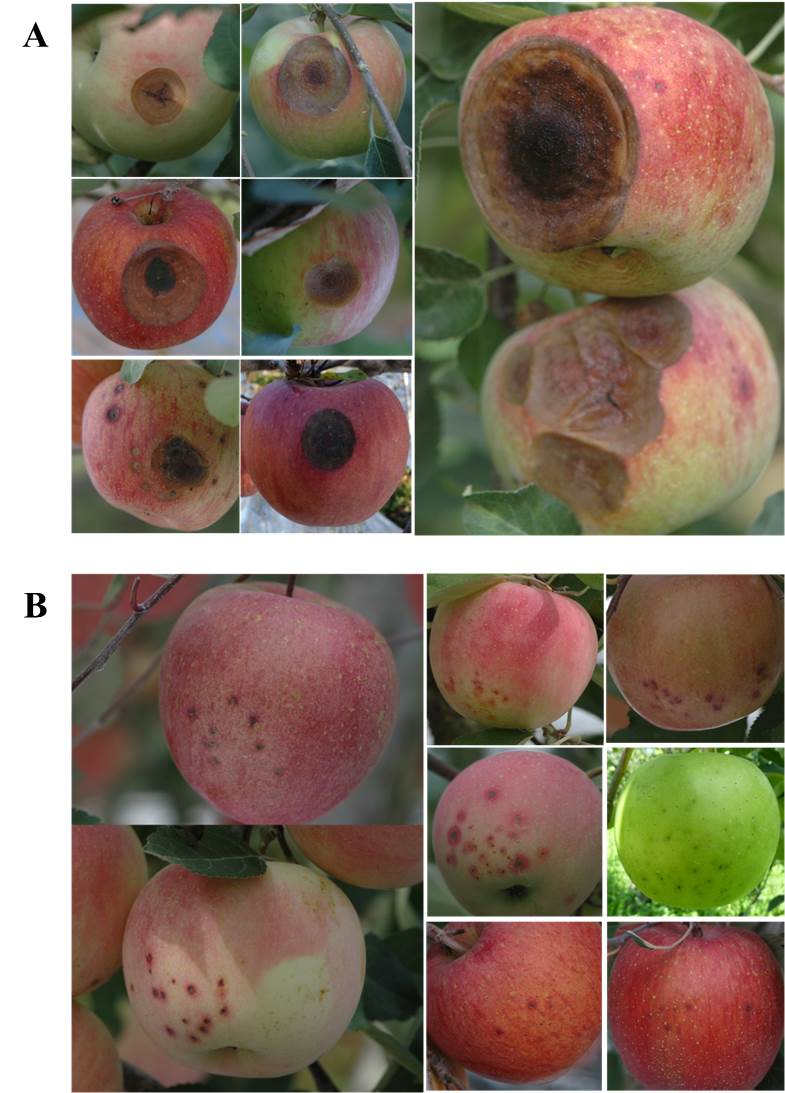


**Fig. S1.** Differentiation of disease symptoms of apple anthracnose caused by *Colletotrichum gloeosporioides* under field conditions. **(a)** Typical progressive symptoms (PS) of anthracnose on apple fruits at different maturity level appear initially as small and sunken, and later enlarged. **(b)** Typical static symptoms (SS) appear as small and dark specks until harvesting season without further enlargement under field conditions.

**
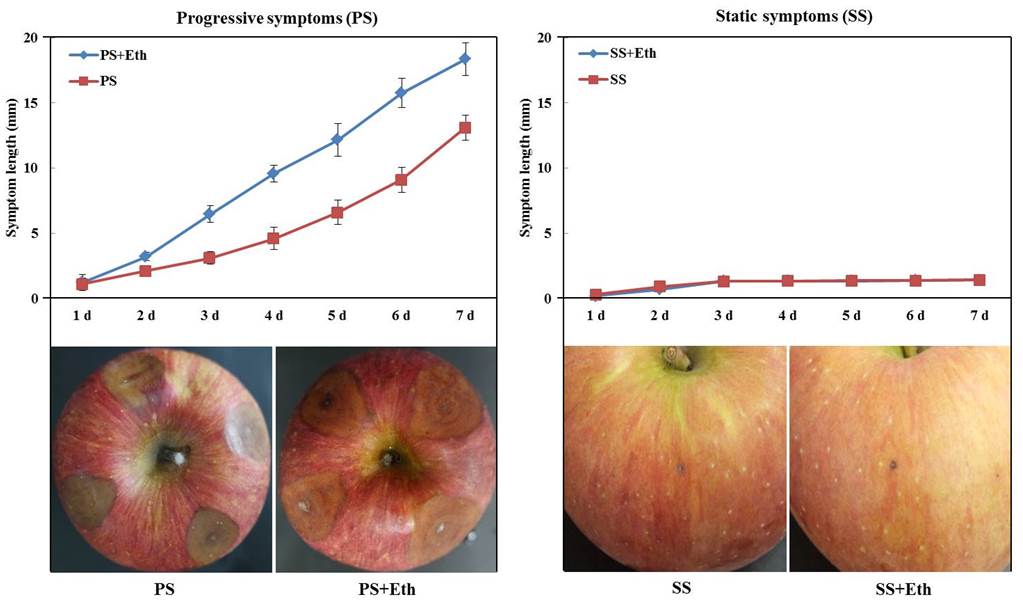
**

**Fig. S2.** Effect of ethephon treatment on progressive symptoms (PS) and static symptoms (SS) of *C. gloeosporioides* to induce disease incidence on ripened apple fruits at different durations. PS with ethephon were more developed than without ethephon treatment, but there was no difference in the development of SS with or without ethephon treatment. Each treatment contained 8 replicates (fruits), and the experiment was carried out at least twice.

**
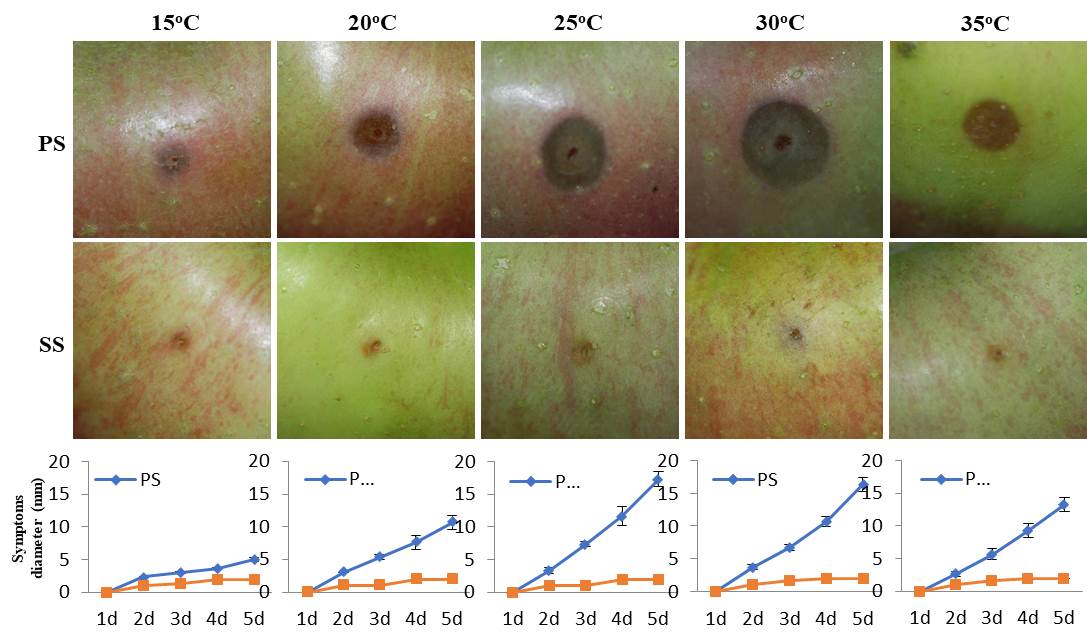
**

**Fig. S3.** Pathogenicity test of both progressive (PS) and static (SS) symptoms on apple fruits at various temperature conditions and different incubation times.

**
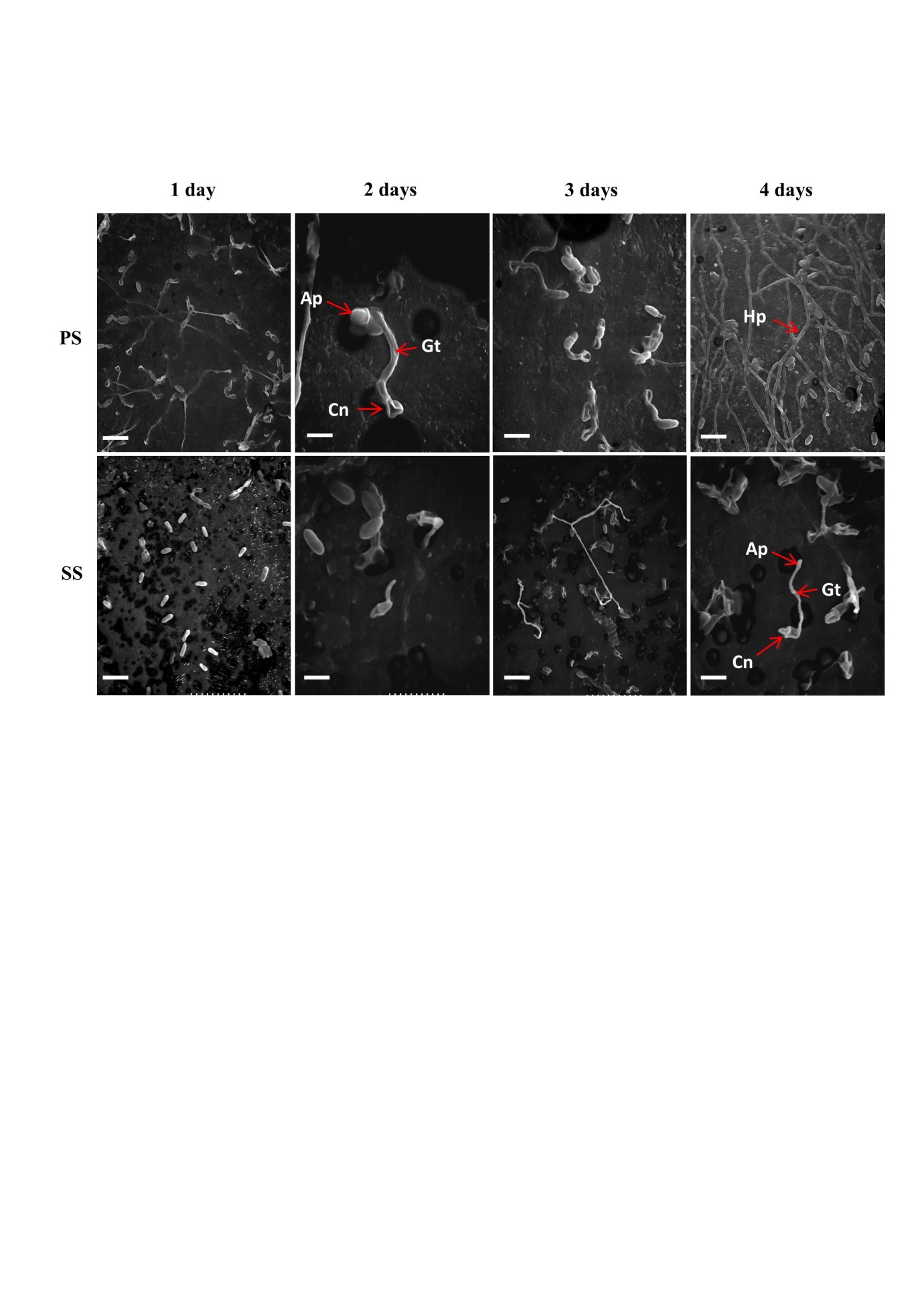
**

**Fig. S4.** Scanning electron microscopic (SEM) analysis of germination and appressorium formation on ripened apple surfaces at different incubation times in both progressive (PS) and static (SS) symptoms of *C. gloeosporioides*. Bar =10 µm. Ap: Appressorium, Cn: Conidium, Gt: Germ tube, Hp: Hyphae, Ph: primary hyphae.

**
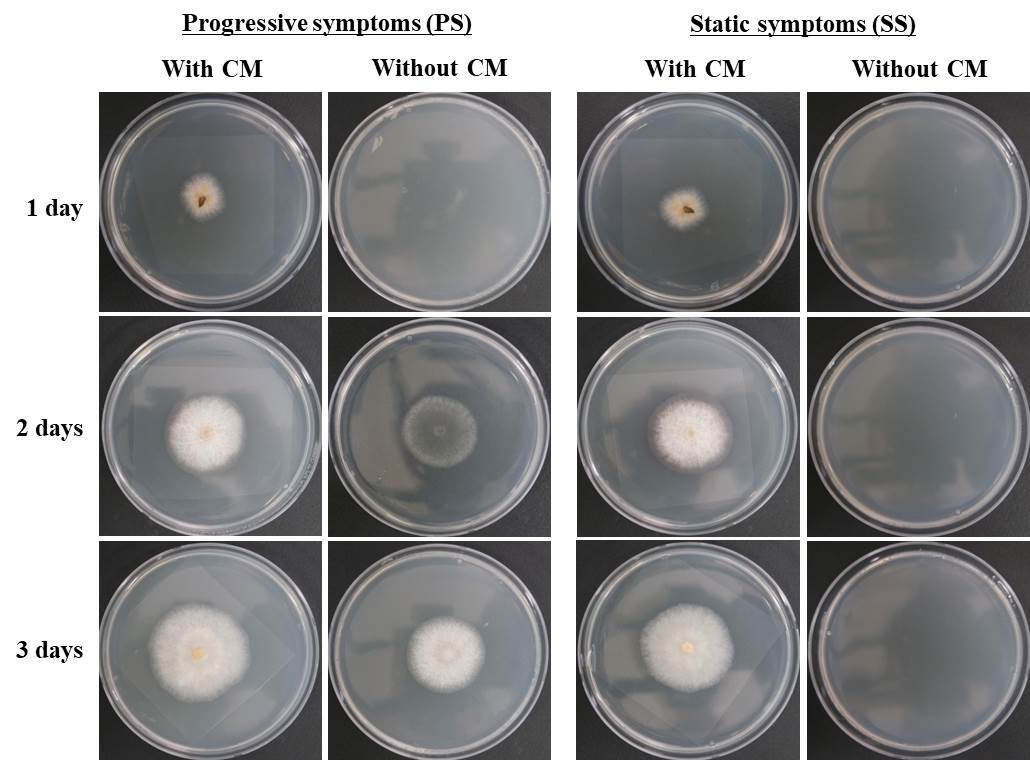
**

**Fig. S5.** Plate assay from water-treated conidia suspensions (10^5^ conidia/mL) of progressive (PS) and static (SS) symptoms of *C. gloeosporioides* from apples on the solid surface of cellophane membranes placed onto PDA plates at various incubation times (1 – 3 days). The plates in the left panel showed mycelial mass of PS on the top of CM, and the conidial germination after removal of CM followed by incubation for 48 h at 25°C. The plates in the right panel showed clear zones after membrane lifting along with the mycelia.


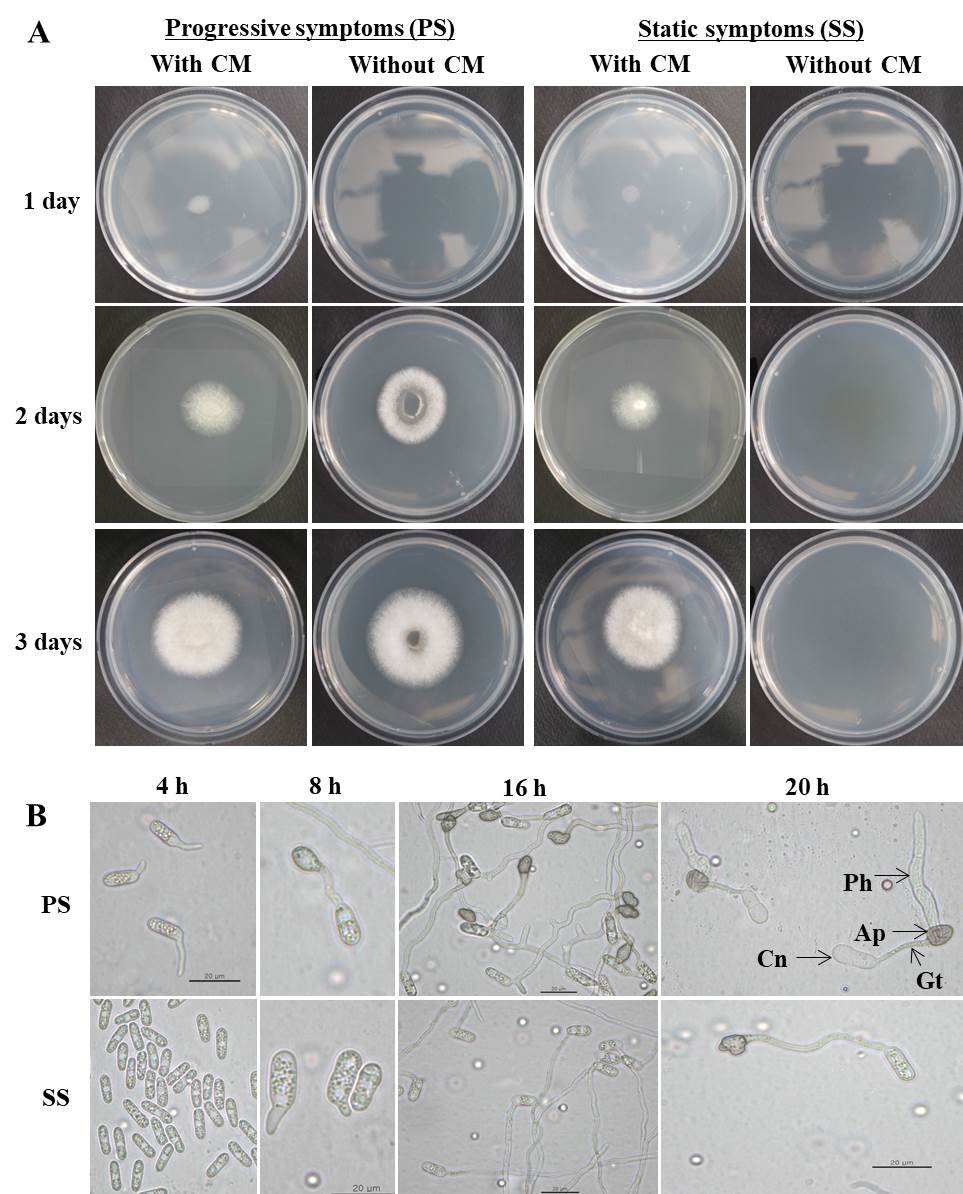


**Fig. S6. Effect of ethephon treatment on mycelial growth of PS and SS of *C. gloeosporioides* and microscopic observations*.* (a)** Plate assay from ethephon-treated conidia suspensions (10^5^ conidia/mL) of PS and SS of *C. gloeosporioides* from apple on the solid surface of cellophane membranes (CM) placed on PDA plates at various incubation times. The plates in the left panel of PS showed mycelial mass of PS on top of CM, and the left panel also shows the conidial germination after removal of CM followed by incubation for 48 h at 25°C. The plates in the right panel of SS showed clear zones after membrane lifting along with the mycelia. **(b)** Microscopic observations showed no effect of ethephon treatment on conidia at 4 h: no germination, 8 h: germination started, 16 h: germination and appressorium formation, and penetration hyphae in SS. However, rapid germination and appressorium formation during or before 20 h in PS. Ap: Appressorium, Cn: Conidium, Gt: Germ tube, Ph: penetration hyphae (infection hyphae).


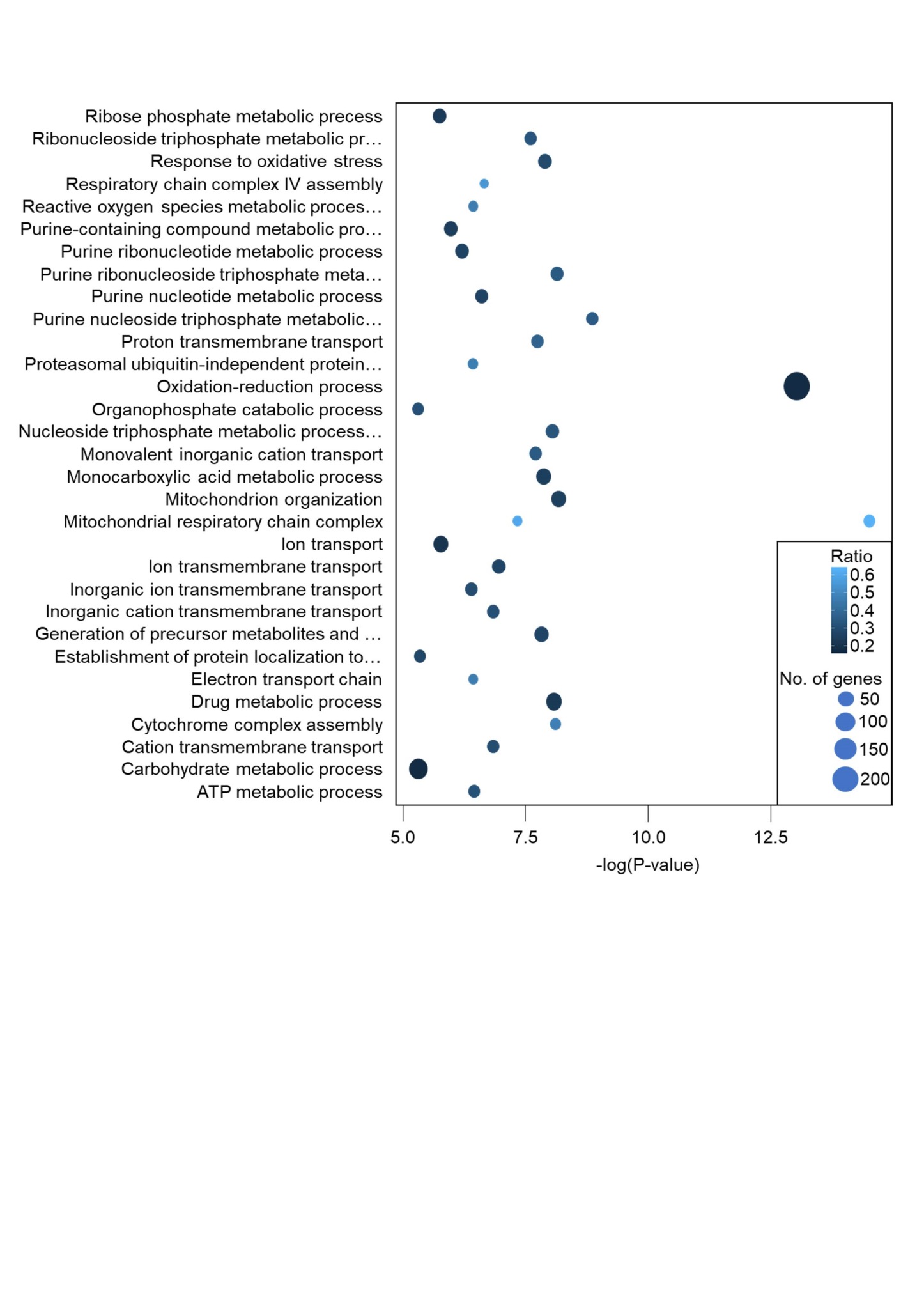


**Fig. S7.** Gene ontology analysis for up-regulated genes associated with biological process categories (PS vs. SS).
